# Inter-platform reproducibility of ultrasound-based fat fraction for evaluating hepatic steatosis in nonalcoholic fatty liver disease

**DOI:** 10.1186/s13244-024-01611-0

**Published:** 2024-02-14

**Authors:** Sun Kyung Jeon, Jeong Min Lee

**Affiliations:** 1 Department of Radiology, Seoul National University Hospital and Seoul National University College of Medicine, 101 Daehangno, Jongno-Gu, Seoul, 03080 South Korea; 2 Institute of Radiation Medicine, Seoul National University Medical Research Center, Seoul, South Korea

**Keywords:** Ultrasonography, Liver, Fatty liver, Nonalcoholic fatty liver disease

## Abstract

**Objectives:**

To evaluate the inter-platform reproducibility of ultrasound-based fat fraction examination in nonalcoholic fatty liver disease (NAFLD).

**Methods:**

Patients suspected of having NAFLD were prospectively enrolled from January 2023. Ultrasound-based fat fraction examinations were performed using two different platforms (ultrasound-derived fat fraction [UDFF] and quantitative ultrasound-derived estimated fat fraction [USFF]) on the same day. The correlation between UDFF and USFF was assessed using Pearson correlation coefficient. Intraclass correlation coefficient (ICC), Bland–Altman analysis with 95% limits of agreement (LOAs), and the coefficient of variation (CV) were used to assess inter-platform reproducibility.

**Results:**

A total of 41 patients (21 men and 20 women; mean age, 53.9 ± 12.6 years) were analyzed. Moderate correlation was observed between UDFF and USFF (Pearson’s *r* = 0.748; 95% confidence interval [CI]: 0.572–0.858). On Bland–Altman analysis, the mean difference between UDFF and USFF values was 1.3% with 95% LOAs ranging from -8.0 to 10.6%. The ICC between UDFF and USFF was 0.842 (95% CI: 0.703–0.916), with a CV of 29.9%.

**Conclusion:**

Substantial inter-platform variability was observed among different ultrasound-based fat fraction examinations. Therefore, it is not appropriate to use ultrasound-based fat fraction values obtained from different vendors interchangeably.

**Critical relevance statement:**

Considering the substantial inter-platform variability in ultrasound-based fat fraction assessments, caution is imperative when interpreting and comparing fat fraction values obtained from different ultrasound platforms in clinical practice.

**Key points:**

• Inter-platform reproducibility of ultrasound-based fat fraction examinations is important for its clinical application.

• Significant variability across different ultrasound-based fat fraction examinations was observed.

• Using ultrasound-based fat fraction values from different vendors interchangeably is not advisable.

**Graphical Abstract:**

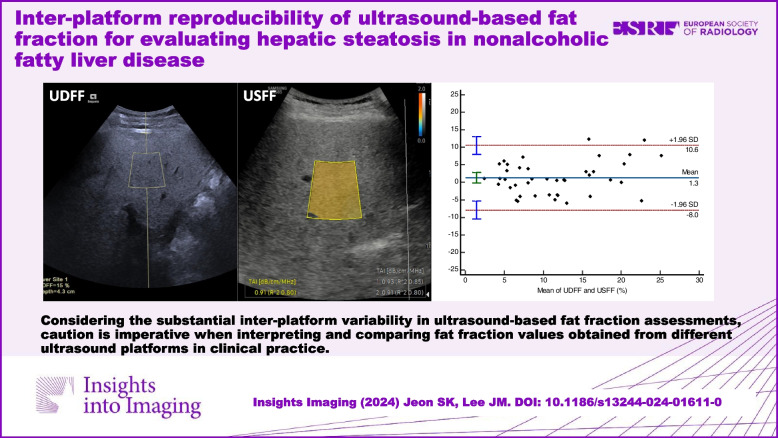

## Introduction

Nonalcoholic fatty liver disease (NAFLD) is identified by the presence of fat accumulation in 5% or more of hepatocytes [1] and is becoming a growing public health concern with increasing prevalence worldwide [2]. NAFLD includes a spectrum of diseases, including isolated hepatic steatosis, nonalcoholic steatohepatitis (NASH), and cirrhosis [3]. Moreover, despite half of the deaths in patients with NASH being attributed to cardiovascular disease or malignancy, awareness remains limited. However, the lack of awareness among patients at risk of progression, along with the absence of a reliable screening method, clarifies why the progression of NASH often remains unnoticed in many cases until cirrhosis has already developed. Early detection and management of hepatic steatosis are known to halt or reverse disease progression [4]. In addition, diagnosing hepatic steatosis and measuring hepatic fat content can be useful for predicting the potential development of cardiovascular diseases or diabetes in the future. However, although there are several potential noninvasive screening tools such as ultrasound (US), blood tests such as fibrosis-4 index, and liver function tests, there is a clinically unmet need for a reliable screening or surveillance modality.

Recently, the development of quantitative ultrasound (QUS) techniques, including attenuation coefficient, backscatter coefficient, and speed of sound, for evaluating of hepatic steatosis has gained significant attention [5, 6]. Despite the potential advantages of QUS techniques, some barriers to their widespread clinical adoption include the presentation of measurement results, reference values, and a lack of standardization among US vendors [7, 8]. A few previous studies have demonstrated the potential of US-based fat fraction for diagnosing and grading hepatic steatosis. Furthermore, the results, presented in the form of percentages, could improve the comprehension of both clinicians and patients [9–12]. Currently, two US-based fat fraction techniques have been successfully commercialized. However, the inter-platform reproducibility of US-based fat fraction values, which is crucial for their clinical application, is still not well established.

Therefore, this study aimed to evaluate the clinical feasibility of US-based fat fraction techniques by assessing the inter-platform reproducibility in patients with NAFLD.

## Methods

This single-center prospective study received approval from our institutional review board, and written informed consent was acquired from all participants.

### Patients

In January 2023, we enrolled 41 participants who met the following eligibility criteria: (1) aged 18 years or older, (2) referred to liver US due to suspected hepatic steatosis, with or without abnormalities in liver function test, and (3) provided written informed consent. We excluded participants with clinical or pathological proof of liver disease except NAFLD, a history of excessive consumption of alcohol (defined as ≥ 14 drinks/week for men and ≥ 7 drinks/week for women), the use of steatogenic or hepatotoxic medications, or a history of hepatic surgery.

### B-mode US and US-based fat fraction examination

#### B-mode US examination

Each participant underwent B-mode US and US-based fat fraction examinations, conducted by one of the two abdominal radiologists (J.M.L. and S.K.J). All participants were instructed to fast for a minimum of 6 h before the examination.

First, a B-mode US examination was conducted with the participant in a supine position, using both the subcostal and intercostal planes. During B-mode liver US examination, the operator assessed the subjective visual score of hepatic steatosis following Hamaguchi’s scoring system as follows: no (0), mild (1), moderate (2), and severe steatosis (3) [13]. Additionally, all stored B-mode US images were independently reviewed by another radiologist who was not involved in the image acquisition, and visual scores of hepatic steatosis were evaluated. Additionally, the distance between the skin and the liver capsule (skin-to-liver capsule distance, in millimeters) was assessed by the operator using the intercostal plane.

#### US-based fat fraction examination

Following the B-mode US examination, the operator performed US-based fat fraction examinations using two different platforms from two different vendors: ultrasound-derived fat fraction (UDFF) using Acuson Sequoia (Siemens Healthineers, Erlangen, Germany) and quantitative ultrasound-derived estimated fat fraction (USFF) using RS 85 (Samsung Medison, Seoul, Korea).

**Fig. 1:**
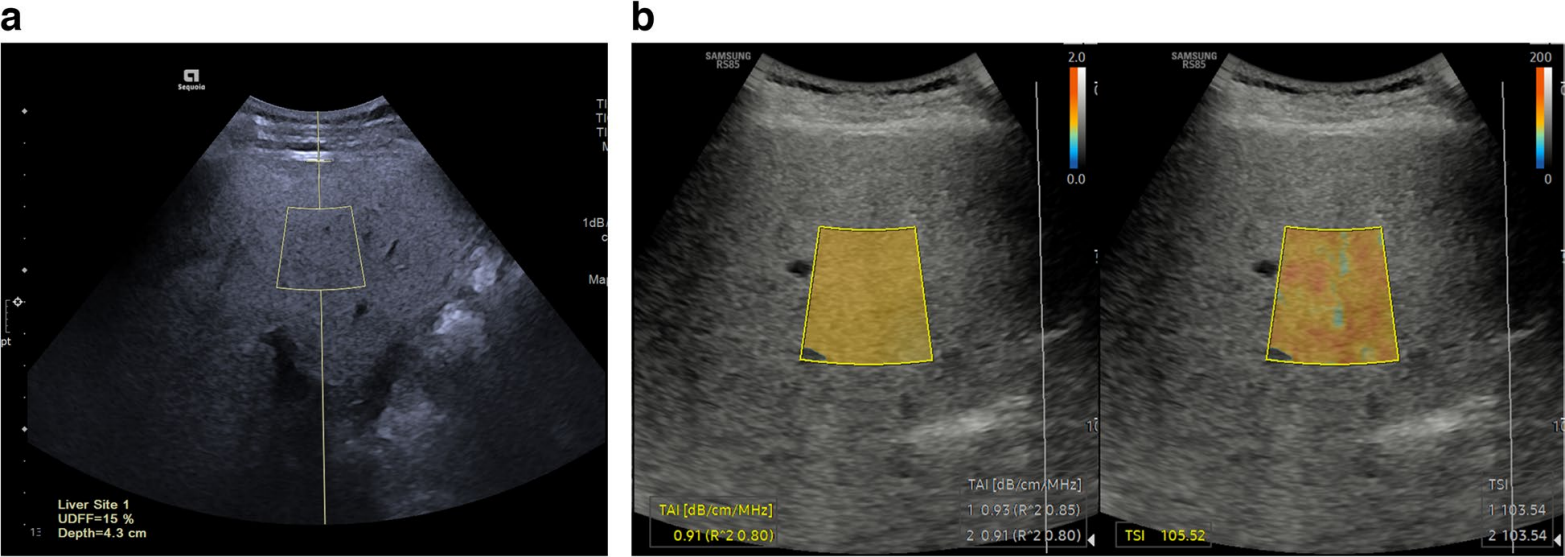
Measurements of ultrasound-based fat fraction measurements using different platforms. Ultrasound-based fat fraction values were measured in each patient using ultrasound-derived fat fraction (UDFF, Siemens Healthineers (**a**)) and quantitative ultrasound-derived estimated fat fraction (USFF, Samsung Medison (**b**))

Measurement of UDFF were performed from the right liver in the intercostal plane using a 2–9-MHz convex probe. The operator placed a rectangular region-of-interest (ROI) box measuring 2.5 cm in length, positioned at least 1.5 cm below the liver capsule. The size and depth of the ROI were predetermined by the manufacturer. The operator conducted five UDFF acquisitions in each session and the median UDFF value obtained from the five acquisitions was used as the value representing each participant according to vendor’s recommendation. Additionally, the operator performed a second session of UDFF acquisition to assess intra-operator inter-session reliability (Fig. 1a).

USFF measurements were performed using a 1–8-MHz convex probe. First, the operator positioned a 2 × 3 cm fan-shaped ROI in the right liver lobe, with a minimum depth of 2 cm below the liver capsule while taking care to avoid areas with reverberation artifacts, focal liver lesions, or large vessels. Tissue attenuation imaging (TAI) values were then automatically calculated. The reliability of each TAI measurement is presented as an *R*^2^ value, and the operator aimed to achieve a TAI value with an *R*^2^ value of at least 0.6 [14]. The tissue scatter-distribution imaging (TSI) value was automatically calculated within the same ROI by selecting a TSI function key. Thereafter, USFF was calculated using the following equation: USFF = -44.3 + 41.9 × TAI + 0.23 × TSI. In each session, the operator conducted five USFF acquisitions, and the mean value derived from these five acquisitions was used as the value representing each participant according to vendor’s recommendation (Fig. 1b).

### Statistical analysis

To compare continuous variables, the independent samples or paired *t*-tests were used, while for comparing categorical variables, the *χ*^2^ test was used. Inter-observer agreement of visual assessment of hepatic steatosis was evaluated using weighted *κ* statistics and interpreted as follows: poor, < 0.20; fair, 0.20–0.39; moderate, 0.40–0.59; substantial, 0.60–0.79; and excellent agreement, > 0.80 [15]. One-way analysis of variance (ANOVA) test following Bonferroni post hot test was used to compare the UDFF or USFF values according to hepatic steatosis grades. Inter-platform reproducibility and inter-session reliability were evaluated using the intraclass correlation coefficients (ICCs). ICC values were interpreted as follows: ≥ 0.90 indicated excellent reliability; 0.75–0.90, good reliability; 0.50–0.75, moderate reliability; and < 0.50, poor reliability [16]. A Bland–Altman analysis with 95% limits of agreement (LOAs) was also performed. The coefficient of variation (CV) was also computed to offer an additional assessment of intra-operator reliability, where a small CV value indicates more reliable measurements [17]. Pearson correlation coefficients were calculated and interpreted using the following criteria: < 0–0.2, indicated very weak; 0.2–0.4, weak; 0.4–0.6, moderate; 0.6–0.8, strong; 0.8–1.0, very strong [18]. To assess the potential impact of patient factors, such as visual hepatic steatosis grades, body mass index (BMI), and skin-to-liver capsule distance on inter-platform variability, Pearson correlation coefficients were analyzed between the absolute differences from different platforms and these factors. Statistical analyses were conducted using commercially available software (MedCalc version 20; MedCalc Software, Mariakerke, Belgium). Statistical significance was defined as a *p* value < 0.05.

## Results

### Study population

Forty-one patients (21 men and 20 women; mean age, 53.9 ± 12.6 years; range, 30–80 years) were enrolled in the study. Table 1 summarizes the demographic characteristics of the study population. The mean BMI was 26.2 ± 3.2 kg/m^2^ (range, 19.6–32.7) and the mean skin-to-liver capsule distance was 19.5 ± 3.7 mm (range, 11–30). On visual assessment, patients were categorized into different steatosis grades: no steatosis (*n* = 4, 9.8%), mild steatosis (*n* = 7, 17.1%), moderate steatosis (*n* = 17, 41.5%), and severe steatosis (*n* = 4, 9.8%). The inter-observer agreement of visual assessment of hepatic steatosis was moderate (*κ* = 0.468; 95% confidence interval [CI] 0.278–0.658).

**Table 1:** Participant characteristics

Characteristic	*n* = 41
Age (years)	53.9 ± 12.6 (30−80)
Sex	
Male	21 (51.2)
Female	20 (48.8)
Body mass index (kg/m^2^)	26.2 ± 3.2 (19.6−32.7)
Skin-to-liver capsule distance (mm)	19.5 ± 3.7 (11–30)
Visual hepatic steatosis grade	
No steatosis (S0)	4 (9.8)
Mild steatosis (S1)	7 (17.1)
Moderate steatosis (S2)	17 (41.5)
Severe steatosis (S3)	4 (9.8)

Values are presented as mean ± standard deviation (range) or number (%), as appropriate

### Inter-platform reproducibility of US-based fat fraction examination

The mean estimated fat fraction values were 12.1 ± 7.1% and 11.0 ± 6.0% for UDFF and USFF, respectively. The mean UDFF and USFF did not show a significant difference (*p* = 0.084). The mean UDFF and USFF values for different hepatic steatosis grades are summarized in Table 2. Both mean values showed significant differences between S1 and S2 and S2 and S3; however, there was no statistically significant difference between S0 and S1.

**Table 2:** US-based fat fraction values according to hepatic steatosis grades

	S0 (*n* = 4)	S1 (*n* = 13)	S2 (*n* = 20)	S3 (*n* = 4)	*p* value	Post hoc analysis		
						S0 vs. S1	S1 vs. S2	S2 vs. S3
UDFF (%) (Siemens)	4.3 ± 1.0	7.3 ± 2.3	14.6 ± 6.0	23.5 ± 4.4	< 0.001	> 0.999	0.001	0.007
USFF (%) (Samsung)	4.1 ± 1.9	6.5 ± 3.8	12.8 ± 3.8	20.9 ± 3.4	< 0.001	> 0.999	< 0.001	0.001

*UDFF*, ultrasound-derived fat fraction (Siemens Healthineers); *USFF*, quantitative ultrasound-derived estimated fat fraction (Samsung Medison)

*p* values were calculated using one-way analysis of variance with Bonferroni post hoc analysis

**Fig. 2:**
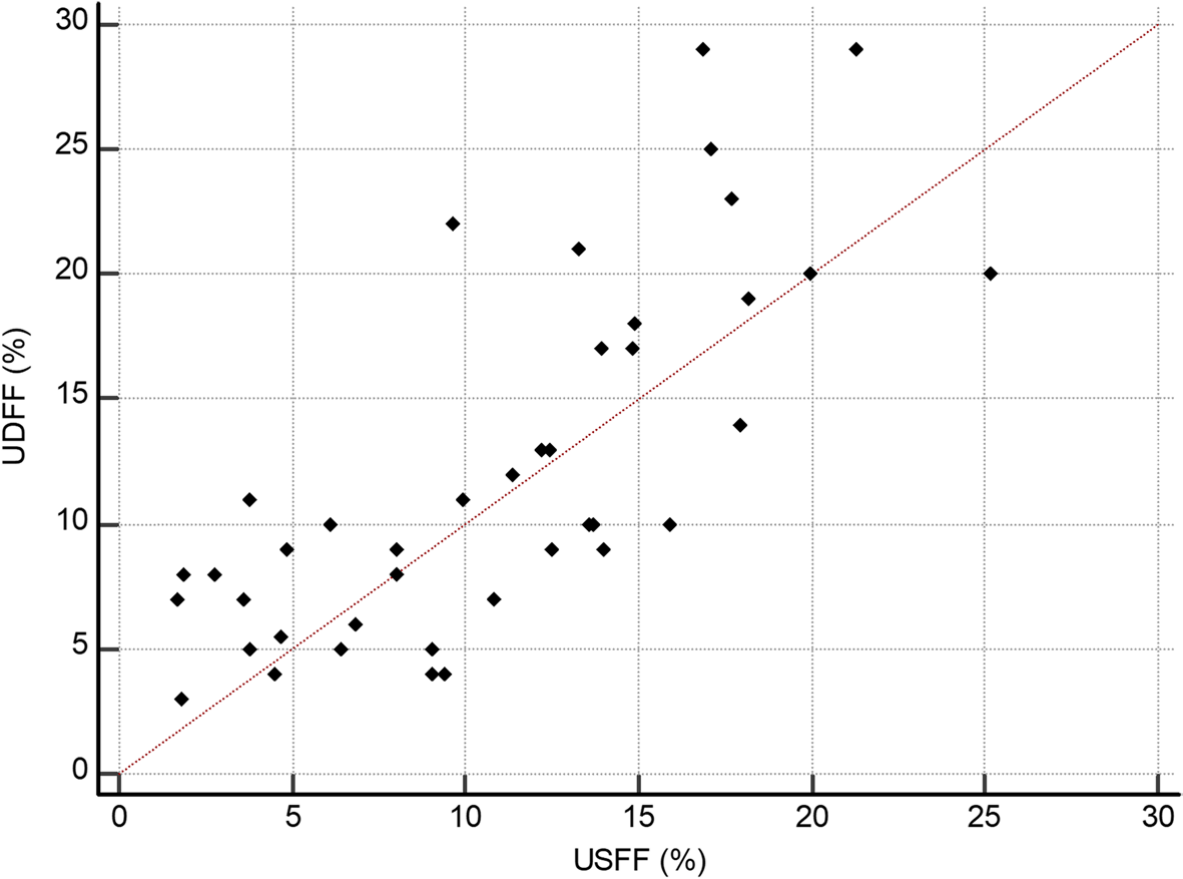
Ultrasound-derived fat fraction (UDFF, Siemens Healthineers) and quantitative ultrasound-derived estimated fat fraction (USFF, Samsung Medison)

**Fig. 3:**
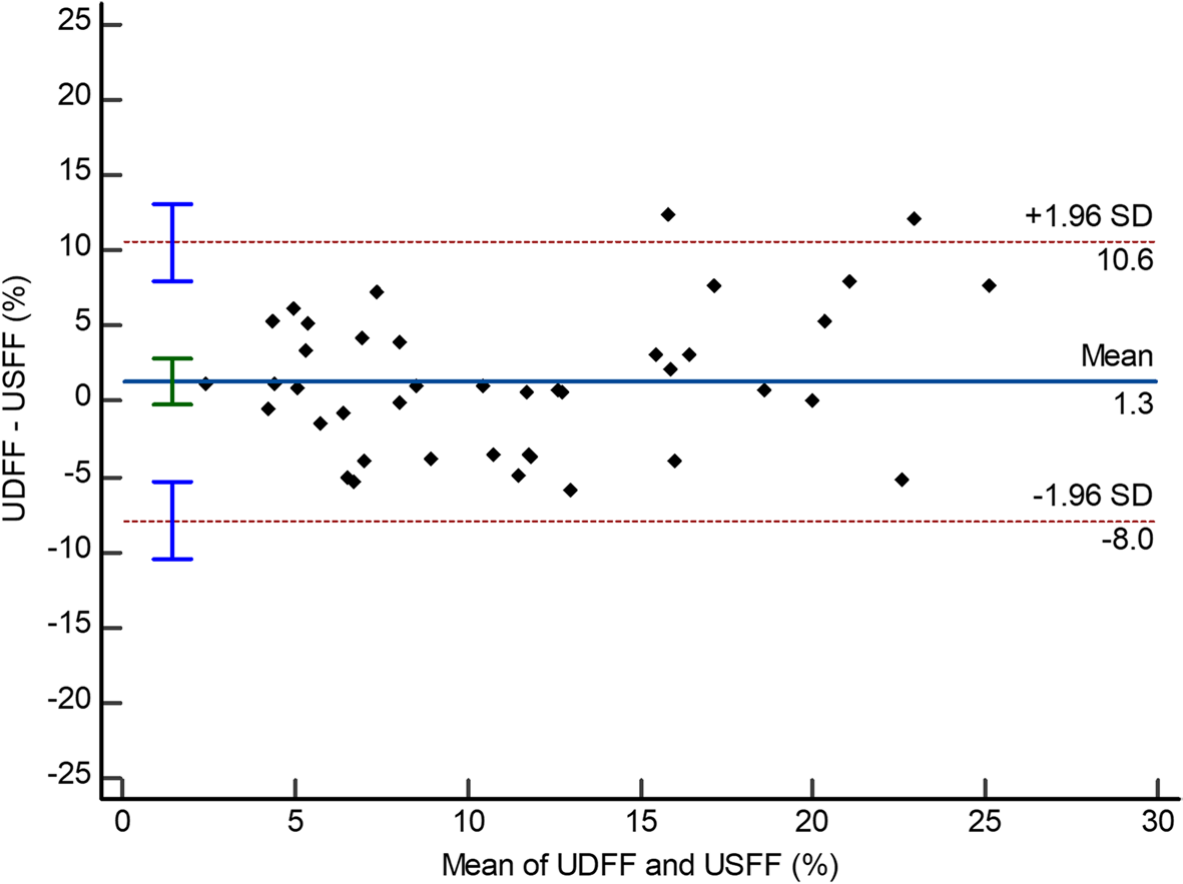
Bland–Altman plots of ultrasound-based fat fraction examination platforms. Bland–Altman plots demonstrated differences in ultrasound-based fat fraction values between the ultrasound-derived fat fraction (UDFF, Siemens Healthineers) and the quantitative ultrasound-derived estimated fat fraction (USFF, Samsung Medison)

The UDFF and USFF values showed a moderate correlation (Pearson *r* = 0.748; 95% CI: 0.572–0.858) (Fig. 2). The ICC of UDFF and USFF was 0.842 (95% CI: 0.703–0.916), with a CV of 29.9%. The Bland–Altman analysis demonstrated a bias between the UDFF and USFF values, with a mean difference between of 1.3% and 95% LOAs ranged from -8.0 to 10.6% (Table 3, Fig. 3).

**Table 3:** Inter-platform reproducibility of US-estimated fat fraction

	Mean bias	BALA	ICC	CV (%)	Pearson *r*
UDFF (Siemens) vs. USFF (Samsung)	1.3 (-0.2, 2.8)	-8.0, 10.6	0.842 (0.703, 0.916)	29.9	0.748 (0.572, 0.858)

Numbers in parentheses are 95% confidence intervals. *UDFF *ultrasound-derived fat fraction (Siemens Healthineers), *USFF* quantitative ultrasound-derived estimated fat fraction (Samsung Medison), *BALA* Bland–Altman 95% limits of agreement, *ICC* intraclass correlation coefficient

Visual hepatic steatosis grade, BMI, and skin-to-liver capsule distance did not correlate with the absolute inter-platform differences in estimated fat fraction (*r* = 0.133, *p* = 0.406 for visual hepatic steatosis grade; *r* = -0.213, *p* = 0.205 for BMI; and *r* = -0.125, *p* = 0.460 for skin-to-liver capsule distance).

### Inter-session reliability of US-derived fat fraction (UDFF)

The inter-session reliability of the UDFF was excellent, with an ICC of 0.963 (95% confidence interval [CI], 0.931–0.980) and CV of 15.3%. The Bland–Altman analysis demonstrated a mean difference of 0.0% with 95% LOAs of the mean estimated fat fraction ranging from -5.2 to 5.2%.

Visual hepatic steatosis grade, BMI, and skin-to-liver capsule distance did not correlate with absolute inter-session differences in UDFF (*r* = -0.176, *p* = 0.272 for visual hepatic steatosis grade; *r* = -0.080, *p* = 0.636 for BMI; and *r* = -0.159, *p* = 0.347 for skin-to-liver capsule distance).

## Discussion

Several previous studies have reported the high diagnostic accuracy of US-based fat fraction in assessing hepatic steatosis, as indicated by the areas under the curve ranging from 0.90 to 0.97 [9–12]. To ensure the wide clinical adoption of US-based fat fraction techniques as surveillance or monitoring tools to evaluate therapeutic responses, assessing inter-platform reproducibility is of great importance. However, the inter-session reliability and inter-platform reproducibility of US-based fat fraction measurements have not yet been fully established. To our knowledge, this is the first study to investigate the inter-platform reproducibility of US-based fat fraction examinations. Our results demonstrated significant inter-platform variability in US-based fat fraction values obtained from different vendors. This variability between the two platforms emphasizes the need for cautious interpretation values derived from different platforms. It is crucial to acknowledge that these values cannot be used interchangeably, particularly in the context of longitudinal follow-ups, where consistent and reliable measurements are essential.

Considering the inter-platform variability of the US-based fat fraction observed in our study, the use of platform-specific cutoff values is necessary for interpreting each US-based fat fraction measurement, and these measurements cannot be used interchangeably. Additionally, in our study, the significant inter-platform variability in US-based fat fraction examinations was not influenced by patient factors such as skin-to-liver capsule distance or BMI. Therefore, the observed variability is likely attributed primarily to factors related to the system and the specific methodology used by the software to calculate US-based fat fractions. Meanwhile, there have been some studies that evaluated inter-platform reproducibility of US attenuation examinations. Previous studies on the inter-platform reproducibility of attenuation coefficient values have reported conflicting results. While some studies have reported good inter-platform reproducibility [19, 20], another study demonstrated substantial inter-platform variability, suggesting notable differences in the obtained values [21]. Furthermore, several studies have reported significant variation in optimal cutoff values of attenuation coefficients obtained from different platforms for diagnosing hepatic steatosis [22–24]. Additionally, previous studies have also demonstrated variations in the results of backscatter coefficient measurements using different methods [7, 8]. As both the UDFF and USFF values were calculated by a multiple logistic regression model using attenuation coefficient and backscatter coefficient, it is not clear whether the attenuation coefficient or backscatter coefficient contributed more significantly to inter-platform variability. Further studies using ideal phantoms are required to confirm this finding.

In our study, UDFF values showed excellent inter-session reliability. Furthermore, the mean bias and 95% LOAs observed in our study were minimal, indicating a small discrepancy between measurements (mean bias, -0.3%; and 95% LOAs, -4.5 to 3.9%). Although the inter-session reliability of US-based fat fraction (USFF) has not been previously investigated, several reports have examined the inter- and intra-examiner reproducibility of attenuation coefficients or backscatter coefficients of QUS. These studies consistently demonstrated the high reproducibility of the attenuation or backscatter coefficients of different platforms, with ICCs ranging from 0.79 to 0.99 [25, 26]. Given the high inter-session reliability of the UDFF, it can be helpful as a screening tool for longitudinal treatment monitoring for hepatic steatosis.

However, an important consideration should be taken into account regarding the clinical use of UDFF. In our study, reliable measurements were obtained in 90.2% of patients using the UDFF, and patient factors such as visual hepatic steatosis grades, BMI, and skin-to-liver capsule distance were not associated with unreliable measurements. However, as our study included a small study population, further investigations with larger study populations are warranted to identify potential factors associated with unreliable measurements and ensure the robustness and reliability of the UDFF in clinical practice.

Our study has several limitations. First, it was a single-center study including a relatively small study population. Further investigations with larger study populations are necessary to validate and generalize our results. Second, our study did not have a reference standard for hepatic steatosis, such as a histological diagnosis or magnetic resonance proton density fat fraction. This was because the primary objective of our study was to assess the inter-platform reproducibility of the US-based fat fraction, rather than directly comparing the diagnostic performance of each platform. Although the absence of a reference standard may limit the ability to draw definitive conclusions about the accuracy of measurements, our study focused on assessing the consistency and reliability of US-based fat fraction measurements across different platforms. Future studies should consider incorporating a reference standard to further evaluate the diagnostic performance of US-based fat fractions in comparison with other established methods. Furthermore, conducting further research that incorporates theses reference standards and explores the inter-platform reproducibility of assessing steatosis grades based on histopathology or MRI-PDFF (not the fat-fraction value itself) can provide substantial clinical significance. Third, the inter-session reliability of the USFF was not evaluated, as previous studies have already reported the inter- and intra-observer reliabilities of the TAI and TSI, which are used to calculate the USFF [25]. However, the good inter-session reliability observed in TAI and TSI cannot be directly extrapolated to assure the inter-session reliability of USFF. Further validation is necessary to assess the inter- and intra-observer reliability of the USFF itself. Fourth, for a more precise assessment of the disease status in patients with NAFLD, a comprehensive interpretation of hepatic steatosis, utilizing clinical-laboratory data or fibrosis stage obtained from transient elastography or shear wave elastography, is essential.

In conclusion, significant inter-platform variability was observed among different US-based fat fraction examinations. Therefore, it is not appropriate to use US-based fat fraction values obtained from different vendors interchangeably.

## Data Availability

The data used to support the findings of this study are available from the corresponding author upon reasonable request.

## Abbreviations

ANOVA: Analysis of variance
BMI: Body mass index
CV: Coefficient of variation
ICC: Intraclass correlation coefficient
IQR: Interquartile range
LOA: Limits of agreement
NAFLD: Nonalcoholic fatty liver disease
NASH: Nonalcoholic steatohepatitis
QUS: Quantitative ultrasound
ROI: Region-of-interest
TAI: Tissue attenuation imaging
TSI: Tissue scatter-distribution imaging
UDFF: Ultrasound-derived fat fraction
USFF: Quantitative ultrasound-derived estimated fat fraction
